# Diacetylcurcumin: Its Potential Antiarthritic Effect on a Freund’s Complete Adjuvant-Induced Murine Model

**DOI:** 10.3390/molecules24142643

**Published:** 2019-07-21

**Authors:** Carolina Escobedo-Martínez, Silvia Laura Guzmán-Gutiérrez, María Isabel Carrillo-López, Martha Alicia Deveze-Álvarez, Alfonso Trujillo-Valdivia, William Meza-Morales, Raúl G. Enríquez

**Affiliations:** 1 Departamento de Farmacia, División de Ciencias Naturales y Exactas, Universidad de Guanajuato, Campus Guanajuato, Guanajuato, Gto. CP 36050, Mexico; 2 CONACyT-Instituto de Investigaciones Biomédicas, Departamento de Inmunología, Universidad Nacional Autónoma de México, CDMX. CP 04510, Mexico; 3 Instituto de Química, Universidad Nacional Autónoma de México, México, CDMX. CP 04510, Mexico

**Keywords:** diacetylcurcumin, curcumin, Freund’s complete adjuvant, antiarthritic

## Abstract

The present study aims to evaluate the antiarthritic activity of diacetylcurcumin (DAC), a synthetic derivative where the free phenolic groups of curcumin are derivatized by acetylation, thereby conferring greater lipophilicity to the parent molecule and partially overcoming the limited systemic bioavailability of curcumin. Antiarthritic activity was evaluated on a Freund’s complete adjuvant (FCA)-induced murine model of arthritis. Oral administration of DAC (60 and 120 mg/kg) resulted in a significant inhibition of inflammation in the acute and chronic phases, respectively, demonstrating an improved and sustained anti-inflammatory effect, comparable to that of curcumin (150 mg/kg) in the chronic stage at a lower dose. Phenylbutazone (80 mg/kg) was used as a reference drug. The pharmacological consequence of DAC or curcumin treatment is the prevention of secondary lesions commonly associated with this biological model.

## 1. Introduction

Inflammation is an essential and protective immune response against microbial invasion, the entrance of antigens, and damage to cells and tissues. This complex process involves different types of cells and pro- and anti-inflammatory mediators that regulate cell migration, chemotaxis, and proliferation in a highly coordinated manner. If acute inflammation fails to control pro-inflammatory stimulus, it leads to chronic inflammation, auto-immunity, and excessive tissue damage [1,2]. 

Rheumatoid arthritis (RA) is an autoimmune disease characterized by chronic inflammation that causes varying degrees of damage to articular cartilage and subchondral bone [3]. RA is the most common cause of physical disability in developed countries, where prevalence ranges between 0.3% and 1.5%, with a 3:1 female-male ratio [4]. Although several medications (steroidal or non-steroidal anti-inflammatory and immunosuppressive drugs) have been used in recent decades to treat RA, more specific drugs with fewer side effects are urgently needed [5,6]. In this regard, plant-derived products are gaining interest since they are generally less toxic, less expensive, and easily accessible sources of new drugs.

Curcumin, a good example of these candidate compounds, has been assayed in experimental and clinic studies to validate its use against several human diseases [7,8].

Curcumin (diferuloylmethane) is the active component of turmeric (*Curcuma longa*), a spice native to India. It is often referred to as a highly pleiotropic molecule with cicatrizing, antimicrobial, immunomodulatory, and antiproliferative activities, being capable of interacting with numerous inflammation-related molecular targets [8,9,10]. Several studies have shown the positive effect of curcumin in the treatment of experimental autoimmune arthritis and animal models of rheumatoid arthritis [11,12,13], incluiding freund’s complete adjuvant model [14].

This botanic dietary component has a potent anti-inflammatory activity, inhibiting all types of inflammation mediators: cytokines, chemokines, adhesion molecules, and growth factors, as well as cyclooxygenase-2 (COX-2), and inducible nitric oxide synthase (iNOS) [15]. These effects of curcumin are due to its capacity to inhibit the pathway of the nuclear factor kappa-light-chain-enhancer of activated B cells (NF-κB) and other proinflammatory signaling pathways, including the Activator protein 1 (AP-1), early growth response protein 1 (Egr-1), and Janus kinase signal transducer and transcription activator (JAK-STAT) [9].

Although there is a remarkably extensive record on the use of curcumin in traditional medicine, it was not considered to be a drug candidate in western medicine until relatively recently [16]. In 1973, Srimal and Dhawan [17] proposed the chemical synthesis of curcumin as a reliable source of research material, noting its lack of toxicity, multifactorial anti-inflammatory action, and very low ulcerogenic index. These desirable traits were highlighted when a possible use against RA was suggested [7], with some advantages over non-steroidal anti-inflammatory drugs (NSAID). However, in the last three decades, studies on animals have demonstrated that curcumin is hydrolytically unstable at intestinal pH levels, it is also rapidly metabolized, conjugated in the liver, and excreted in feces [8]. Thus, the systemic bioavailability of curcumin is limited by intestinal and hepatic glucuronidation as a part of its metabolism [16], and different derivatives and analogs have been prepared over time to overcome this major drawback [18,19].

Diacetylcurcumin (DAC) is a synthetic derivative of curcumin where phenolic OH groups are protected with acetyl groups. This results in increased lipophilicity, possibly leading to a higher bio-membrane penetration rate. DAC has been reported to have important biological properties, e.g., high antibacterial activity and anti-biofilm activity against methicillin-resistant *Staphylococcus aureus* strains [20] and antimalarial activity in vitro against chloroquine-resistant *Plasmodium falciparum* [21]. DAC has potential as an antiproliferative agent in anticancer therapies [22]. Thus, studies on molecular human colon cancer cells (HCT116) have shown that DAC prevents the correct formation of the mitotic spindle, hindering chromosome alignment and, therefore, blocking cells in mitosis [23]. In addition, there are controversial studies on the effect of DAC observed on carrageenan-induced inflammation, ranging from a total absence of activity [24] up to a maximum value of activity in inhibition of edema, followed only by aspirin and curcumin [25], including a potent anti-inflammatory effect on par with other curcumin analogues [26]. Thus, we report herein for the first time the antiarthritic activity of DAC on a RA murine model.

## 2. Results

### 2.1. Acute Toxicity Studies

No CD1 mice mortality was observed in the groups orally receiving curcumin or DAC at doses up to 5000 mg/kg. The body weight of treated CD1 mice increased gradually with time, with no significant differences with respect to the control group since the first day of treatment (Data not shown).

### 2.2. Anti-Inflammatory Activity

#### 2.2.1. Behavior of Controls

**Figure 1:**
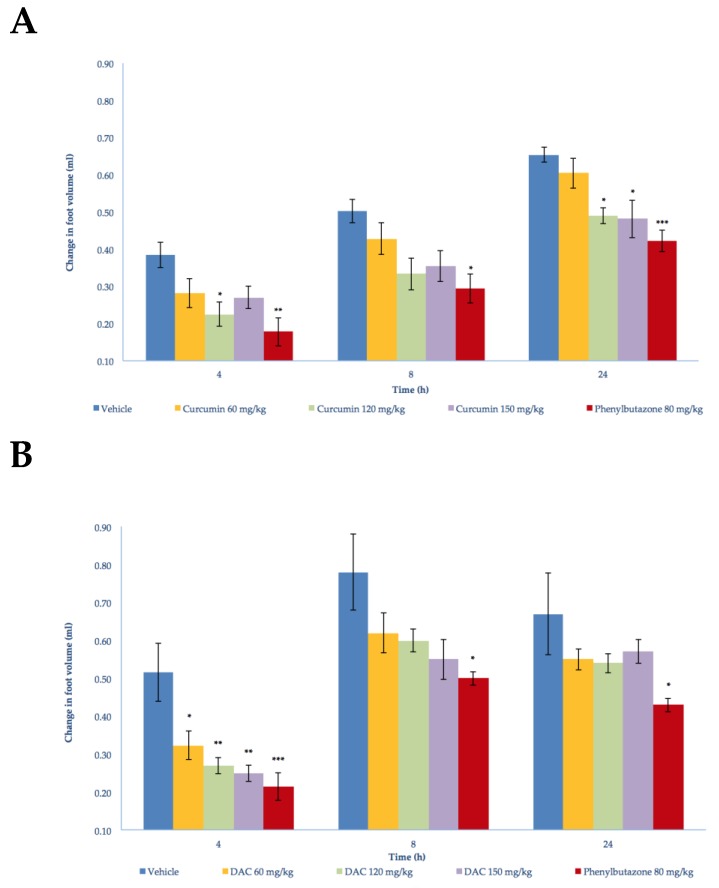
Acute effect of the oral administration of phenylbutazone (80 mg/kg), (**A**) curcumin, and (**B**) diacetylcurcumin (DAC) (60, 120, and 150 mg/kg) on the edema produced by intradermal injection of Freund’s complete adjuvant into the footpad of right rear limbs of Wistar rats. Each bar represents the mean ± SEM (*n* = 5). ANOVA followed by Tukey’s test. *p* < 0.05, 0.01 and 0.001 (*, ** and *** respectively).

**Figure 2:**
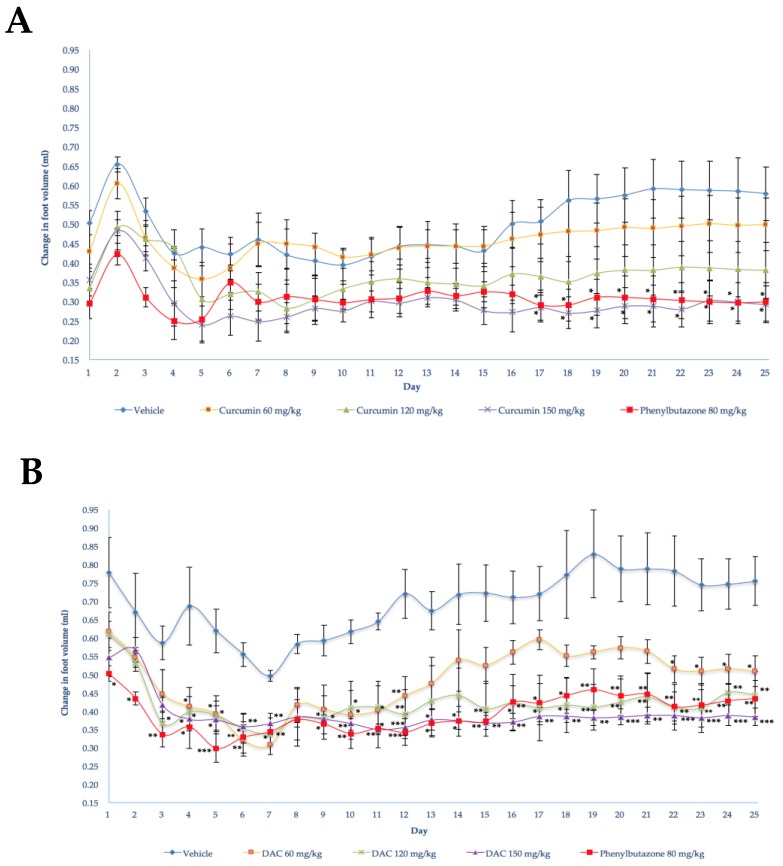
Antiinflammatory effect during the chronic phase of the oral administration of phenylbutazone (80 mg/kg), (**A**) curcumin, and (**B**) diacetylcurcumin (DAC) (60, 120, and 150 mg/kg) upon the edema induced by intradermal injection of Freund’s complete adjuvant into the footpad of right rear limbs of Wistar rats. Each point represents the mean ± SEM (*n* = 5). ANOVA followed by Tukey’s test. *p* < 0.05, 0.01 and 0.001 (*, ** and *** respectively).

The antiarthritic activity of DAC and curcumin was assessed in independent experiments. The Freund’s complete adjuvant (FCA) injection in the footpad of the right rear limb of rats in the control group (vehicle) used in the curcumin experiment caused an edema that reached its maximum volume within the acute phase, that is, the first 3–5 days [27] (Figure 1A). In the following days, the edema showed a sudden increase on day 2 and decreased slowly from day 3 to day 10. On day 11, the edema showed a small increase, which became marked after day 15, increasing and reaching its maximum value on days 20–25 (Figure 2A). During this last period, such values were superimposed with those of the period of edema formation in the chronic phase, emulating an arthritic status.

In the DAC control group experiment (using a vehicle), the maximum edema was observed 8 h after FCA injection (Figure 1B). The edema decreased slowly until day 3, exhibiting a sudden increase on day 4 and a marked and continuous decrease until day 7. After day 8, a daily and significant increase of the edema was observed. The edema reached its highest value on days 18–22, showing a small decrease until the end of the experiment (Figure 2B). These results are similar to those obtained in the curcumin experiment for the chronic phase. The positive controls using phenylbutazone in both experiments showed anti-edema activity (80 mg/Kg) throughout the acute phase (Figure 1A,B) and chronic phase. This substance has been previously used in other models as a suitable reference drug to inhibit the formation of edema caused by CFA injection in the hind paws of rats [28,29].

#### 2.2.2. Effect of the Oral Administration of the Diacetylcurcumin and Curcumin against FCA-Induced Arthritis

Continuous measurements of the footpad of the right rear limb of the animals with a plethysmometer demonstrated that treatment with phenylbutazone, curcumin, or DAC at 60, 120, and 150 mg/kg doses inhibited the formation of the edema during the period under study. During the acute phase of the edema formation (Figure 1), the treatment with curcumin showed a significant inhibitory effect at 4 h (120 mg/kg) and 24 h (120 mg/kg and 150 mg/kg) after FCA injection. Similar findings were observed in the group treated with DAC at all doses (60, 120, and 150 mg/kg), which significantly inhibited the formation of the edema during the acute phase by 37.21%, 47.67%, and 51.55%, respectively (Figure 1, see Supplementary Material). The reference drug (phenylbutazone, 80 mg/kg) significantly inhibited the edema at 4, 8, and 24 h in both experiments.

During the chronic phase, the oral administration of curcumin caused a significant inhibition of the edema on days 17–25 (150 mg/kg) (Figure 2A). Compared with phenylbutazone, the curcumin showed a slight increase in the percentages of edema inhibition. Furthermore, the administration of DAC caused a significant decrease in the formation of the edema at different doses on days 4, 5, 6, 7, 10, 11, and 12 (60 mg/kg); on days 3, 4, 5, 6, 7, 9, 11, 12, and 15–25 (120 mg/kg); and on days 4, 5, 6, and 9–25 (150 mg/kg) (Figure 2B). In the chronic phase (after day 16), administration of DAC at 120 mg/kg led to inhibition percentages that were equal to, or surpassed, those caused by the administration of phenylbutazone.

**Figure 3:**
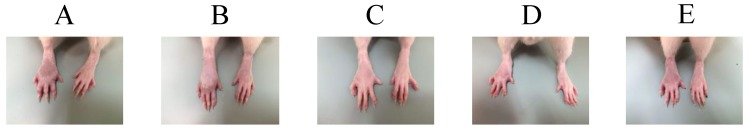
Edema status after oral treatment (on day 25) with curcumin at different doses. (**A**) Vehicle, (**B**) Curcumin 60 mg/kg, (**C**) Curcumin 120 mg/kg, (**D**) Curcumin 150 mg/kg, and Phenylbutazone (80 mg/kg) (**E**).

**Figure 4:**
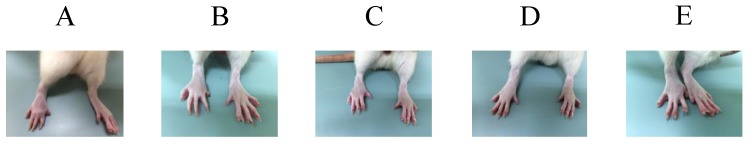
Edema status after oral treatment (on day 25) with diacetylcurcumin (DAC) at different doses. (**A**) Vehicle, (**B**) DAC 60 mg/kg, (**C**) DAC 120 mg/kg, (**D**) DAC 150 mg/kg and Phenylbutazone (80 mg/kg) (**E**).

The oral administration of DAC at doses of 120 and 150 mg/kg led to earlier, more sustained, and more efficacious anti-edema activity (on days 3–25 and 4–25, respectively) than curcumin at a dose of 150 mg/kg, with a significant reduction of swelling after day 17 until the end of the experiment (Figure 2B). Visually, the footpads of rats treated with DAC at doses of 120 mg/kg and 150 mg/kg were clearly healthier than the footpads of rats receiving curcumin. During the whole experiment, no secondary lesions were observed in the non-injected footpads of the left rear limbs in any of the groups treated with curcumin, DAC, or phenylbutazone (Figure 3 and Figure 4). Additionally, all treated groups showed acceptable joint mobility. The mean body weight showed no significant difference (on day 25) between that of the animals receiving curcumin or DAC orally at any dose, and that of the group treated with the vehicle. However, a loss in body weight seems to be a trait of the inflammation model itself [30].

## 3. Discussion

To assess the anti-arthritic effect of DAC in this study, one of the most important pharmacological models was used. The arthritic status was induced by an injection of a dispersion of heat-killed and freeze-dried mycobacteria into the footpad of the right rear limb of rats. 

Although the pathogenesis of adjuvant arthritis is not clearly understood, there is a good amount of evidence indicating that this disease is the result of an aberrant immune response. The humoral immune response also plays a very important role in the initial phase of the disease [31]. On the other hand, arthritis due to FCA injection at the footpad of the right rear limb of rats caused the primary lesion, characterized by inflammation at the application site, while secondary lesions resulted in the swelling of the footpad of the left rear limb and other parts of the body, such as ears or tail. There is an alternative method to induce arthritis in a murine model, based on a single FCA injection at the base of the tail. However, this method is considered unsatisfactory to cause arthritis [32].

Our results showed that groups administered with the vehicle showed the peak of the chronic phase to occur between days 18 and 21 [32]. Thus, it is safe to assume that the arthritic status existed in all FCA-administered groups. Therefore, our results, suggesting a significant inhibition of inflammation at the doses tested, are trustworthy. On the other hand, the reference drug phenylbutazone, used herein at a lower dose than the 100 mg/kg reported by Perper et al. [32], was efficacious, providing significant protection against primary and secondary lesions (Figure 3 and Figure 4).

In the acute phase, observed 4 h after FCA injection, DAC showed better results against inflammation than curcumin. Even at the lowest dose administered (60 mg/kg), DAC caused a significant edema inhibition percentage (37.21%). In contrast, curcumin required a higher dose (120 mg/kg) to cause a significant decrease of 41.67% in the edema. This fact is probably due to the acetylation of the phenolic groups of the precursor molecule curcumin, resulting in increased lipophilicity of the molecule and probably increased penetration in the gastrointestinal membrane.

During the chronic phase (days 17–25), the administration of curcumin (150 mg/kg) led to inhibition percentages slightly higher than the reference drug: 43.9–52.4% and 42.7–49.3%, respectively (Figure 2, see Supplementary Material). On the other hand, DAC administration resulted in a significant inhibition of edema after day 4 at any dose. The best results were obtained at day 17, when DAC inhibited the edema by 43.33–47.57% and 46.39–51.27% at doses of 120 and 150 mg/kg, respectively. These effects were better than those of phenylbutazone (41.39–47.31%) in the same period (Figure 2, see Supplementary Material). These results demonstrate the improved and sustained anti-inflammatory effect of DAC, similar to curcumin at a dose of 150 mg/kg, but the effect of DAC starts at lower doses. This action is probably due to the acetylation of the phenolic hydroxyl groups of the precursor molecule, curcumin, which have increased the lipophilicity of the molecule. Consequently, the greater penetration of the gastrointestinal membrane, like that which happened in *in vitro* studies when evaluating the antibacterial activity of DAC and curcumin, shows that DAC has action at much lower concentrations [20].

While non-steroidal anti-inflammatory drugs (NSAIDs) like aspirin, indomethacin, and naproxen have been widely used to treat arthritis symptoms, they have been found to cause severe irritation in the gastric mucosa after extended use [33,34]. This is due to the common mechanism of action of NSAIDs, the inhibition of the cyclooxygenase known as prostaglandin-endoperoxide synthase (PGH synthase), a speed-limiting enzyme that converts arachidonic acid into prostaglandins (PG). Based on this, curcumin has been proven to be a biologically safe and efficient source material for research, since its lack of toxicity, anti-inflammatory action, and very low ulcerogenic index have been established [17]. These traits have been highlighted to propose its use against RA [7], with various advantages over NSAIDs. The results reported here indicate that the curcumin analogue, DAC, is an even more efficacious anti-inflammatory drug than its parent compound and also has a shorter response time. On the other hand, DAC is practically non-toxic, allowing an oral intake of 5 g/kg. Moreover, DAC accumulation after a daily administration of the maximum dose (150 mg/kg) for 14 days did not result in any signs of toxicity during the period under study. Thus, our results support the anti-inflammatory potential of this curcumin analogue. Furthermore, because curcumin is a major metabolite that naturally occurs in the plant species *Curcuma longa* and DAC is prepared by synthetic modification of curcumin, both compounds were obtained with a high degree of purity, as shown by high-performance liquid chromatography (HPLC) and nuclear magnetic resonance (NMR) and demonstrated by their usefulness as pharmacological therapeutic agents in a model of rheumatoid arthritis.

## 4. Materials and Methods

### 4.1. Drugs and Chemicals

Phenylbutazone and Freund’s complete adjuvant (FCA) were purchased from Sigma (St. Louis, MO, USA). Standard curcumin was purchased from ChromaDex (Irvine, CA, USA). Diacetylcurcumin (DAC) was synthetized at the Instituto de Química, UNAM (Mexico City, Mexico). *Curcuma longa* (Turmeric) was purchased from a local market in downtown Mexico City.

### 4.2. Extraction and Purification of Curcumin

Fresh rhizomes were cleaned, washed and sliced. Dried and ground *C. longa* (700 g) was macerated with various solvent consecutive times. The first extraction was performed with dichloromethane, followed by ethyl acetate and methanol, and, later, the extracts were evaporated. The percentages and weights of the curcuminoid extracts were: Dichloromethane 4.3% (30.1 g), ethyl acetate 4.5% (31.5 g), and methanol 5.6% (39.2 g). The methanol extract was subjected to column chromatography in a silica gel (70–150 mesh) glass column. Chloroform and methanol solvent mixtures were used as the mobile phase. All the collected fractions were monitored by thin-layer chromatography. Curcumin was identified by running standard curcumin along with the samples. Crude curcumin was recrystallized in isopropanol three times in a concentration of 6 g/L.

### 4.3. Synthesis of Diacetylcurcumin

Curcumin (5 g, 13.57 mmol) was dissolved in 70 mL of dichloromethane (CH_2_Cl_2_) with 2.20 mL (27.14 mmol) of pyridine. This solution was kept under agitation for 15 min, followed by the dropwise addition of acetic anhydride (1.4 mL, 13.57 mmol). The reaction mixture was kept under agitation for 3 h at room temperature. Then, CH_2_Cl_2_ was evaporated and the residue was extracted with ethyl acetate (3 × 60 mL). The organic phase was dried over anhydrous sodium sulfate and evaporated under reduced pressure. The resultant solid was recrystallized in ethyl acetate to yield yellow crystals (4.2861 g of DAC, mp. 170.2 °C, yield 69.79%).

### 4.4. HPLC and NMR Analysis

**Figure 5:**
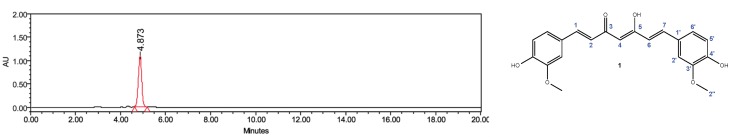
Chromatogram of the curcumin (1) (t_R_ = 4.873 min) sample, injected at a concentration of 0.25 mg/mL, with detection at a wavelength of 265 nm.

**Figure 6:**
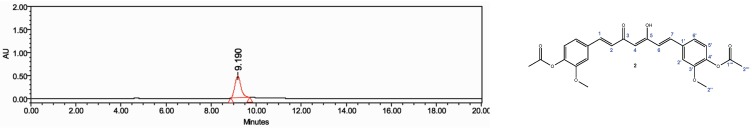
Chromatogram of diacetylcurcumin (2) (t_R_ = 9.190 min) samples, injected at a concentration of 0.25 mg/mL, with detection at a wavelength of 257 nm.

The purity of curcumin (Figure 5) and DAC (Figure 6) was determined by high-performance liquid chromatography (HPLC) in a Waters 1525 Binary HPLC pump equipment, controlled with the Breeze 2 HPLC software (Waters Corporation, Milford, MA, USA). A 100–5 C18 Kromasil column 15 cm × 4.6 mm i.d. and a diode array detector series 2998 (Waters Corporation, Milford, MA, USA), the maximum absorption for curcumin and DAC was set at 265 and 257 nm, respectively) were used. An acetonitrile-methanol-water-acetic acid (2%) isocratic gradient 40:20:40 was applied for 20 min at a flow speed of 1 mL/min.

The nuclear magnetic resonance (NMR) spectra of DAC and curcumin were acquired in Bruker Avance III HD 500 MHz NMR Spectrometer (Bruker Scientific LLC, Billerica, MA, USA) equipment for ^1^H and ^13^C in CDCl_3_ solution, using tetramethylsilane (TMS) as an internal standard (Table 1 and Table 2). NMR spectra were processed with the software MestReNova version: 12.0.0 (Mertrelab Research, S.L., Santiago de Compostela, España) and can be seen in the Supplementary Material. The chemical shift values for DAC were compared with those reported in the literature [35].

### 4.5. Pharmacological Effect

#### 4.5.1. Animals

All experiments were performed in accordance with the applicable ethical guidelines for avoiding pain on experimental research animals [36] and the Mexican Official Norm for animal care and handling (NOM-062-ZOO-1999). The experimental protocol (CIBIUG-P15-2018) and the study were approved and supervised by the Institutional Committee of Ethics for the Care and Use of Laboratory Animals, Universidad de Guanajuato. Inbred Wistar male rats (250–300 g, 7–8 weeks of age) and CD1 male mice (20–25 g), obtained from the vivarium of the Division of Natural and Exact Sciences, Universidad de Guanajuato, were used. All Wistar rats were housed in groups of five (*n =* 5) under controlled temperature (23 ± 2 °C) and humidity (55 ± 10%) conditions, with a 12 h light/darkness cycle, and were familiarized to the environment one week before the experiments. The animals were allowed food and water ad libitum. Immediately after the experiments were completed, all animals were euthanized in a CO_2_ chamber.

#### 4.5.2. Acute Toxicity Studies

The mean lethal dose (LD_50_) by oral route (p.o.) was determined on CD1 male mice, as reported by Lorke [37]. The drug concentration (DAC or curcumin) was adjusted to 3000 and 5000 mg/kg, and administered p.o. (cannula 18G × 1.5″) with a volume of administration of 0.1 mL/10 g. Mice were observed daily for 14 days, recording mortality, toxic effects, and/or weight changes, as well as behavior patterns.

#### 4.5.3. Anti-Inflammatory Activity

The evaluation the effect of curcumin and DAC was done in independent experiments, and 25 rats were required for each experiment (Five groups per experiment: Vehicle, phenylbutazone, and DAC or curcumin (at 60, 120 and 150 mg/kg). The arthritis syndrome was induced by the intradermal injection of 0.05 mL of FCA into the footpad of the right rear limb [28] of Wistar rats using a 20-gauge needle.

The edema evolution was determined by the volume displacement method using a digital plethysmometer (Panlab, Barcelona, Spain). Volume displacement was recorded 24 h before FCA injection and 4, 8, and 24 h after FCA injection. To complete the evaluation of drug activity on arthritis induction, any variation in volume displacement at the footpad of the right rear limb of the subject rats was determined daily until 25 days after injection. The reference drug (phenylbutazone, 80 mg/kg), curcumin, and DAC (at a dose of 60, 120, or 150 mg/kg) were administered p.o. (cannula 16G × 3″) 1 h before FCA injection and for 14 days after. The carrier vehicle used was corn oil in a maximum administration volume of 0.1 mL/100 g.

The percentage of edema inhibition was calculated as the average measurement for each group (*n* = 5) treated, with respect to the average measurement of the group only administered with the vehicle. Differences between the controls and treatments were analyzed with Tukey’s test. A *p*-value less than 0.05 was considered statistically significant. The data were processed with the statistics software GraphPad PRISM 7.0b (GraphPad Software, San Diego, CA, USA).

## 5. Conclusions

The oral administration of diacetylcurcumin (60 mg/kg and 120 mg/kg) caused a significant inhibition of inflammation during the acute and chronic phases, respectively, demonstrating an enhanced and sustained anti-inflammatory effect, surpassing that of curcumin (150 mg/kg) in the chronic stage, and showing a high therapeutic potential as an antiarthritic agent with fewer side effects (low ulcerogenic index). This study supports the traditional use of *Curcuma longa* to treat inflammatory conditions and the positive consequences that chemical modifications may have in improving its therapeutic potential.

## Figures and Tables

**Table 1:** ^1^H-NMR spectral data of curcumin and diacetylcurcumin (DAC) (500 MHz) ^a^.

^1^H ^a^	Curcumin				Diacetylcurcumin			
	Chemical Shift	Multiplicity	Integral	Coupling Constant (Hz)	Chemical Shift	Multiplicity	Integral	Coupling Constant (Hz)
CH_3_-2‴		s	6		2.33	s	6	
CH_3_-2″	3.93	s	6		3.88	s	6	
H-4	5.82	s	1		5.85	s	1	
H-2/H-6	6.48	d	2	*J* = 15.72	6.56	d	2	*J* = 15.86
H-5′	6.91	d	2	*J* = 8.01	7.06	d	2	*J* = 8.11
H-2’/H-6′	7.07	m	4					
H-2′					7.12	d	2	*J* = 1.81
H-6′					7.15	dd	2	*J* = 8.15, 1.82
H-1/H-7	7.57	d	2	*J* = 15.72	7.61	d	2	*J* = 15.78
O-H_aryl_	8.04	Wide signal	2					
O-H	16.11	Wide signal	1		15.85	Wide signal	1	

^a^ Data recorded in CDCl_3_. Chemical shifts (δ) are expressed in ppm with respect to tetramethylsilane (TMS). Assigned coupling patterns: s = singlet, d = doublet, dd = doublet of doublets, m = multiplet. All assignments were derived from experiments: ^1^H-^1^H, ^13^C-^1^H, HSQC (heteronuclear single quantum coherence; couplings ^1^H-^13^C to a bond) and HMBC (heteronuclear multiple bond correlation; couplings ^1^H-^13^C to 2 and 3 bonds). See Supplementary Material.

**Table 2:** ^13^C-NMR spectral data of curcumin and diacetylcurcumin (DAC) (125.7 MHz) ^a^.

Carbon ^a^	Chemical Shift	
	Curcumin	Diacetylcurcumin
C-2‴		20.87
C-2″	55.42	56.11
C-4	100.62	102.01
C-2′	109.66	111.63
C-5′	114.99	123.48
C-2 and C-6	120.75	124.45
C-6′	122.40	121.27
C-1′	126.55	134.15
C-1 and C-7	140.17	140.14
C-4′	147.12	141.49
C-3′	148.31	151.59
C-1‴		169.00
C-3 and C-5	182.76	183.28

^a^ Data recorded in CDCl_3_. Chemical shifts (δ) are expressed in ppm with respect to tetramethylsilane (TMS). All assignments were derived from experiments: ^1^H-^1^H, ^13^C-^1^H, HSQC (heteronuclear single quantum coherence; couplings ^1^H-^13^C to a bond) and HMBC (heteronuclear multiple bond correlation; couplings ^1^H-^13^C to 2 and 3 bonds) 2D NMR spectra. See Supplementary Material.

## Supplementary Materials

The following are available online. Table S1: The effect of curcumin and DAC on the edema induced by Freund’s complete adjuvant on a murine model (acute phase); Table S2: The effect of curcumin and DAC on the edema induced by Freund’s complete adjuvant on a murine model (chronic phase); Figure S1: ^1^H-NMR of curcumin (**1**); Figure S2: ^13^C-NMR of curcumin (**1**); Figure S3: HSQC of curcumin (**1**); Figure S4: HMBC of curcumin (**1**); Figure S5: HMBC of curcumin (**1**); Figure S6: HMBC of curcumin (**1**); Figure S7: ^1^H-NMR of dicaetylcurcumin (**2**); Figure S8: ^13^C-NMR of diacetylcurcumin (**2**); Figure S9: HSQC of diacetylcurcumin (**2**); Figure S10: HSQC of diacetylcurcumin (**2**); Figure S11: HMBC of diacetylcurcumin (**2**); Figure S12: HMBC of diacetylcurcumin (**2**).
