# Supplementary material for: Diacetylcurcumin: Its Potential Antiarthritic Effect on a Freund’s Complete Adjuvant-Induced Murine Model
Source: Molecules. 2019 Jul 21;24(14):2643. doi: 10.3390/molecules24142643 (PMC6680498; doi:10.3390/molecules24142643)
Supplement: Supplementary file 1 [file molecules-24-02643-s001.pdf]

Supplementary Material

Diacetylcurcumin: Its potential antiarthritic effect on a Freund´s complete adjuvant-induced murine model

Carolina Escobedo-Martínez [1,*], Silvia Laura Guzmán-Gutiérrez [2], María Isabel Carrillo-López [1], Martha Alicia Deveze-Álvarez[1], Alfonso Trujillo-Valdivia[1] , William Meza-Morales [3] and Raúl G. Enríquez [3,*]

[1] Departamento de Farmacia, División de Ciencias Naturales y Exactas. Universidad de Guanajuato, Campus Guanajuato. Guanajuato, Gto. CP 36050. México; c.escobedo@ugto.mx
[2] CONACyT - Instituto de Investigaciones Biomédicas. Departamento de Inmunología. Universidad Nacional Autónoma de México, CDMX. CP 04510. México; laura.guzman@iibiomedicas.unam.mx
[3] Instituto de Química, Universidad Nacional Autónoma de México, México, CDMX. CP 04510. México; habib@unam.mx

* Correspondence: c.escobedo@ugto.mx, enriquezhabib@gmail.com

Table S1. The effect of curcumin and DAC on the edema induced by Freund's complete adjuvant on a murine model (acute phase).

Time (hours)	Dose (mg/kg)	Sample	% Inhibition ± SEM	Sample	% Inhibition ± SEM
4	80	Phenylbutazone	**53.65 ±0.04****	Phenylbutazone	**58.14±0.04*****
4	60	Curcumin	26.56±0.04	DAC	**37.21±0.04***
4	120	Curcumin	**41.67±0.03***	DAC	**47.67±0.02****
4	150	Curcumin	29.69±0.03	DAC	**51.55±0.02****
8	80	Phenylbutazone	**41.43±0.04***	Phenylbutazone	**35.48±0.02***
8	60	Curcumin	14.74±0.04	DAC	20.82±0.05
8	120	Curcumin	33.47±0.04	DAC	21.85±0.03
8	150	Curcumin	29.48±0.4	DAC	29.82±0.05
24	80	Phenylbutazone	**35.47±0.03*****	Phenylbutazone	**35.22±0.02***
24	60	Curcumin	7.65±0.04	DAC	18.51±0.03
24	120	Curcumin	**25.08±0.02***	DAC	19.10±0.03
24	150	Curcumin	**26.30±0.05***	DAC	15.22±0.03

[*] Data were analyzed by ANOVA followed by Tukey's test. $P < 0.05$, 0.01 and 0.001 (*, ** and *** respectively).

Table S2. The effect of curcumin and DAC on the edema induced by Freund's complete adjuvant on a murine model (chronic phase).

Time (days)	Dose (mg/kg)	Curcumin experiment	% Inhibition ± SEM	Diacetylcurcumin experiment	% Inhibition ± SEM
17	80	Phenylbutazone	**42.7±0.04***	Phenylbutazone	**41.39±0.05***
17	60	Curcumin	6.7±0.04	DAC	17.50±0-02
17	120	Curcumin	28.1±0.05	DAC	**43.33±0.09***
17	150	Curcumin	**43.9±0.07***	DAC	**46.39±0.03****
18	80	Phenylbutazone	47.29±0.04	Phenylbutazone	**42.75±0.05***
18	60	Curcumin	20.27±0.04	DAC	28.76±0.02
18	120	Curcumin	37.83±0.06	DAC	**46.11±0.08***
18	150	Curcumin	**51.35±.07***	DAC	**50.00±0.03****
19	80	Phenylbutazone	**45.0±0.05***	Phenylbutazone	**44.04±0.06****
19	60	Curcumin	14.5±0.05	DAC	31.87±0.02
19	120	Curcumin	34.0±0.07	DAC	**45.99±0.06****
19	150	Curcumin	**51.1±0.07***	DAC	**50.85±0.02****
20	80	Phenylbutazone	**46.0±0.05***	Phenylbutazone	**43.91±0.05****
20	60	Curcumin	14.6±0.05	DAC	27.41±0.02
20	120	Curcumin	33.8±0.07	DAC	**46.19±0.06****
20	150	Curcumin	**49.8±0.07***	DAC	**51.27±0.03*****
21	80	Phenylbutazone	**48.1±0.06***	Phenylbutazone	**43.40±0.06****
21	60	Curcumin	17.3±0.05	DAC	28.68±0.02
21	120	Curcumin	35.6±0.06	DAC	**44.42±0.07****
21	150	Curcumin	**51.2±0.07***	DAC	**50.76±0.03****
22	80	Phenylbutazone	**48.6±0.05***	Phenylbutazone	**47.31±0.04****
22	60	Curcumin	16.0±0.05	DAC	**34.27±0.02***
22	120	Curcumin	34.0±0.07	DAC	**47.57±0.06****
22	150	Curcumin	**52.4±0.08***	DAC	**50.38±0.04*****
23	80	Phenylbutazone	**49.1±0.05***	Phenylbutazone	**44.09±0.04****
23	60	Curcumin	14.7±0.05	DAC	**31.72±0.03***
23	120	Curcumin	34.1±0.07	DAC	**45.16±0.07****
23	150	Curcumin	**48.5±0.07***	DAC	**48.66±0.04*****
24	80	Phenylbutazone	**49.3±0.05***	Phenylbutazone	**42.63±0.05****
24	60	Curcumin	15.1±0.05	DAC	**31.10±0.03***
24	120	Curcumin	34.6±0.07	DAC	**39.68±0.07****
24	150	Curcumin	**49.0±0.07***	DAC	**47.99±0.04*****
25	80	Phenylbutazone	**48.4±0.05***	Phenylbutazone	**42.44±0.05****
25	60	Curcumin	13.8±0.05	DAC	**32.63±0.02***
25	120	Curcumin	34.3±0.07	DAC	**41.11±0.06****
25	150	Curcumin	**49.5±0.07***	DAC	**49.07±0.04*****

* Data were analyzed by ANOVA followed by Tukey's test. $P < 0.05$, 0.01 and 0.001 (*, ** and *** respectively).

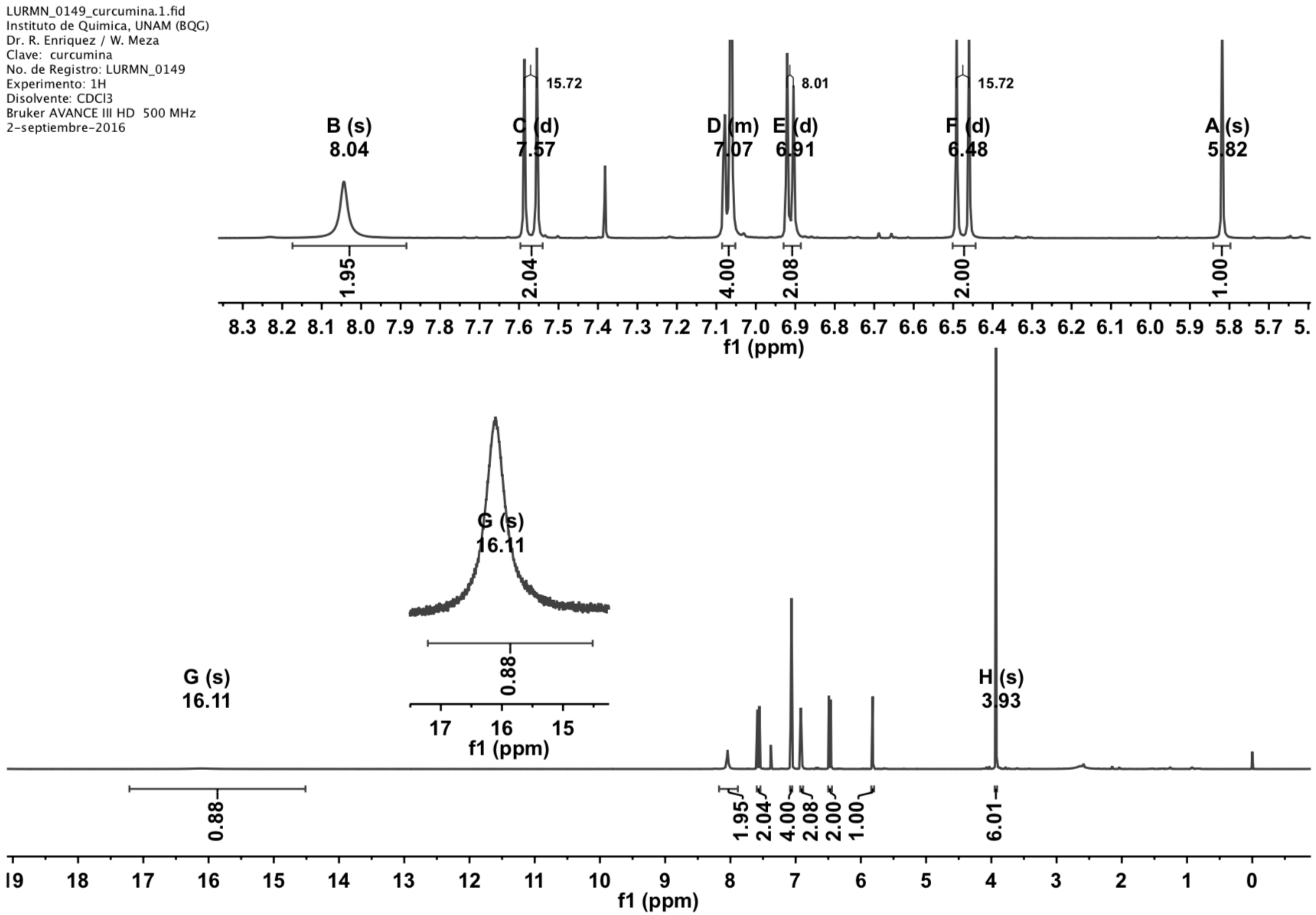

Figure S1: ^{1}H-NMR of curcumin (**1**).

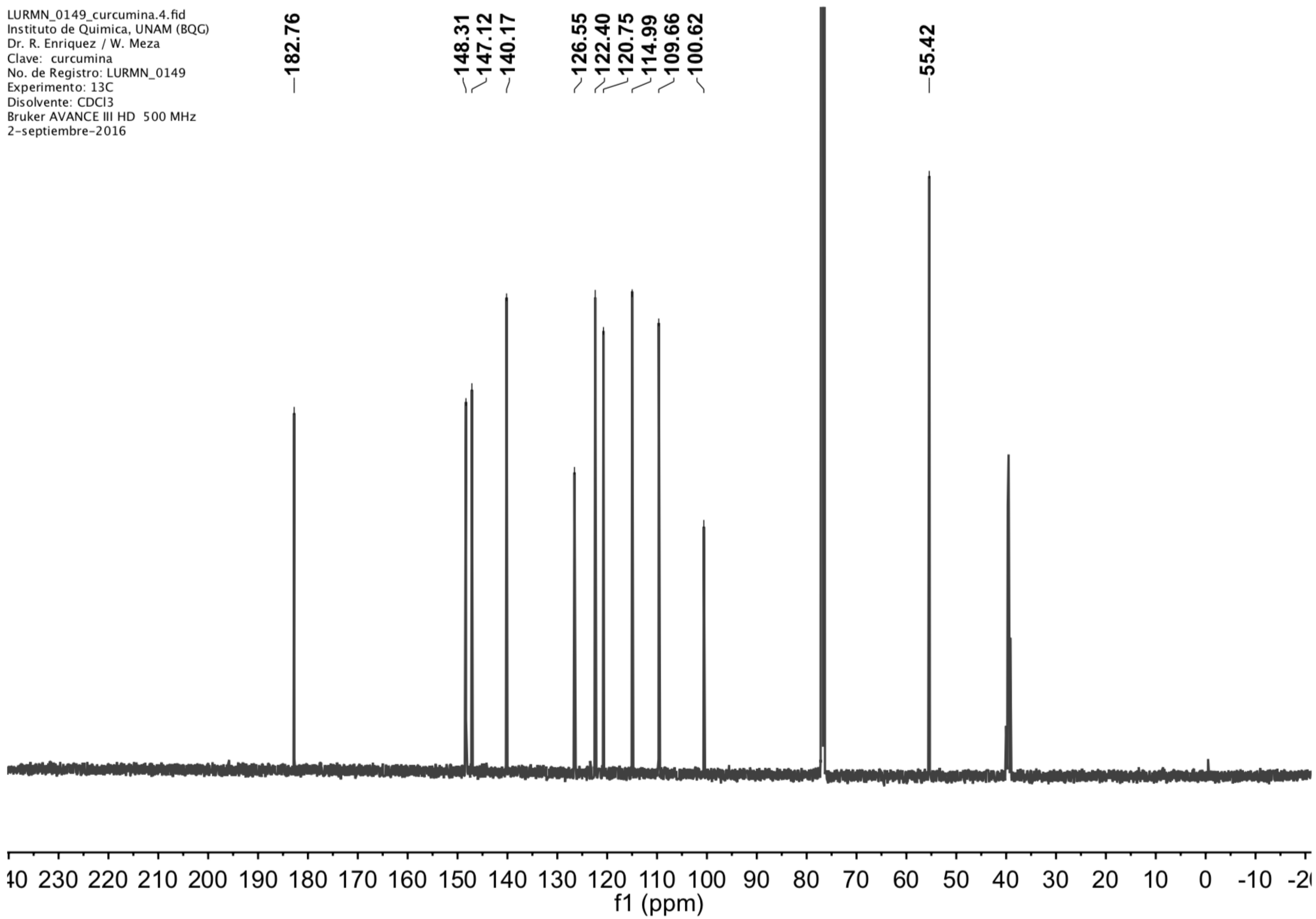

Figure S2: 13C-NMR of curcumin (1).

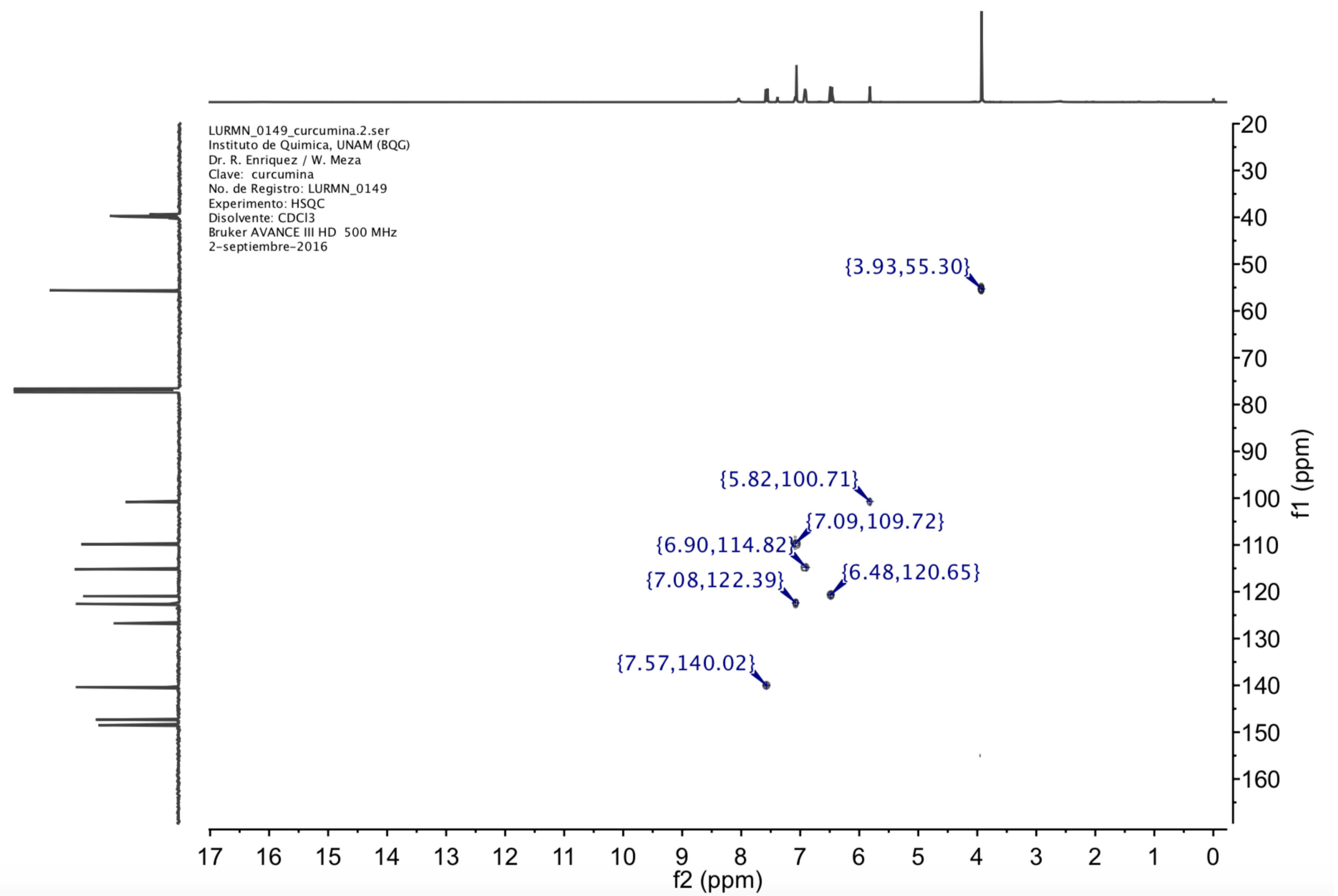

Figure S3: HSQC of curcumin (**1**).

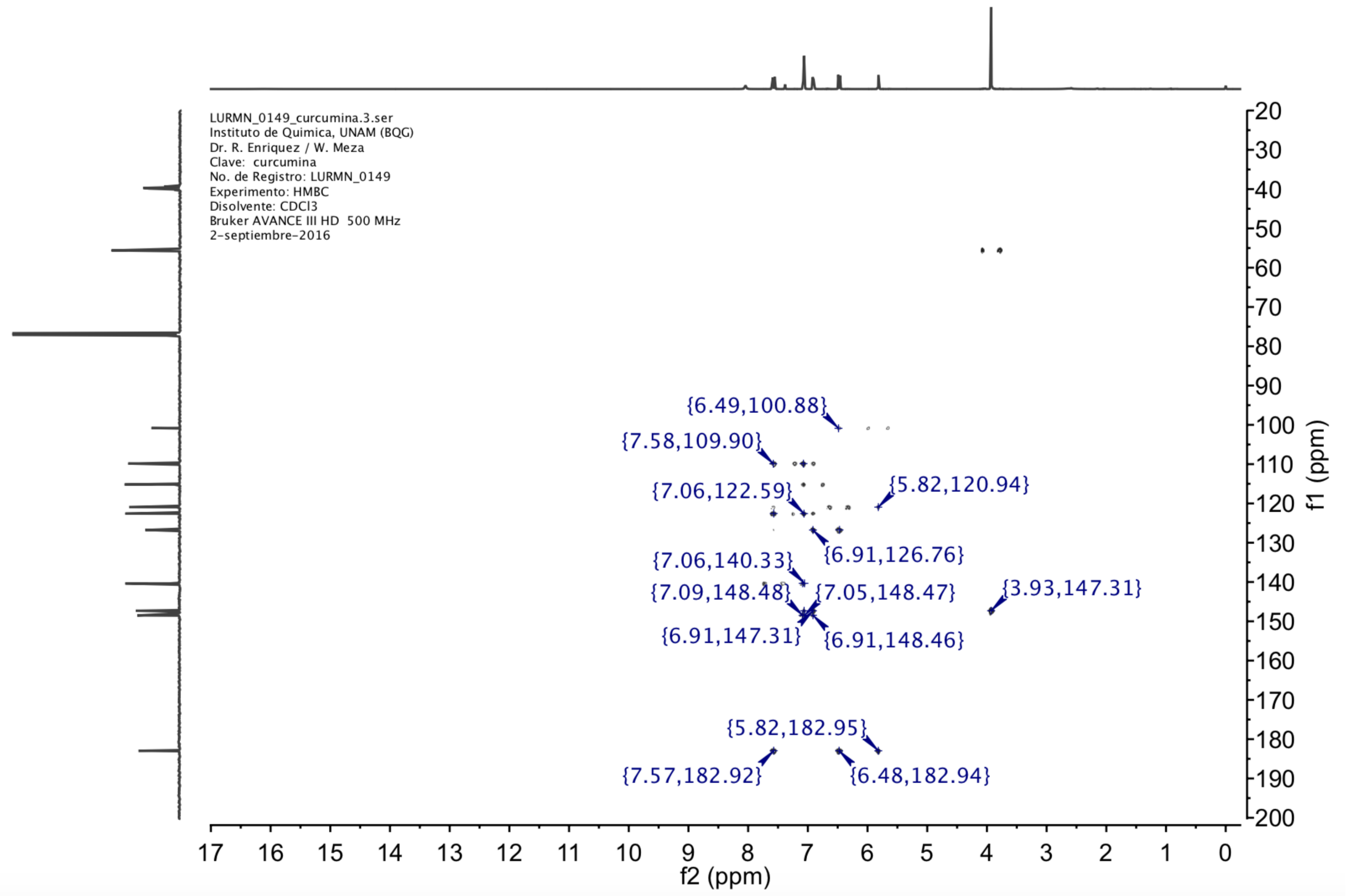

Figure S4: HMBC of curcumin (**1**).

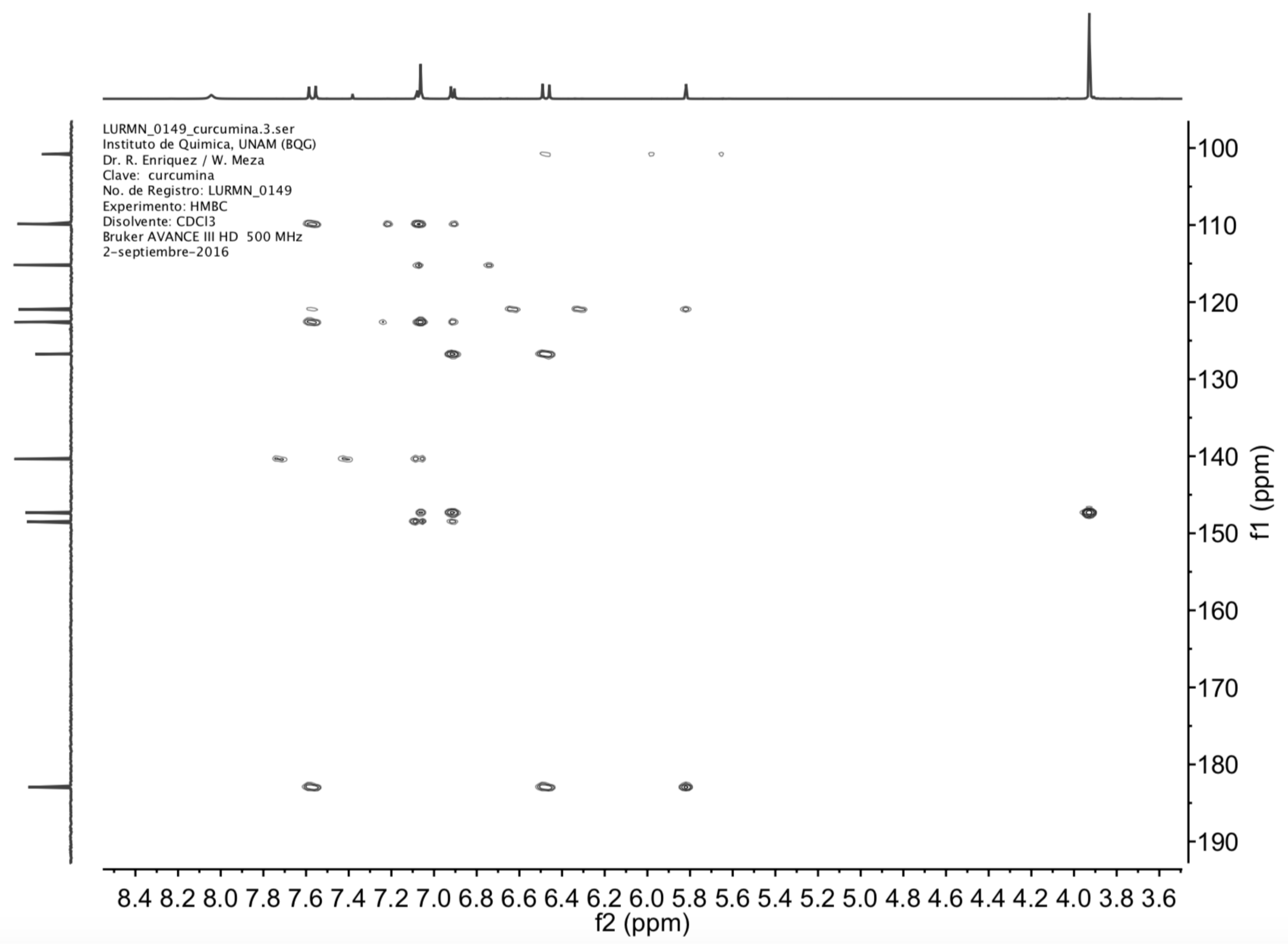

Figure S5: HMBC of curcumin (**1**).

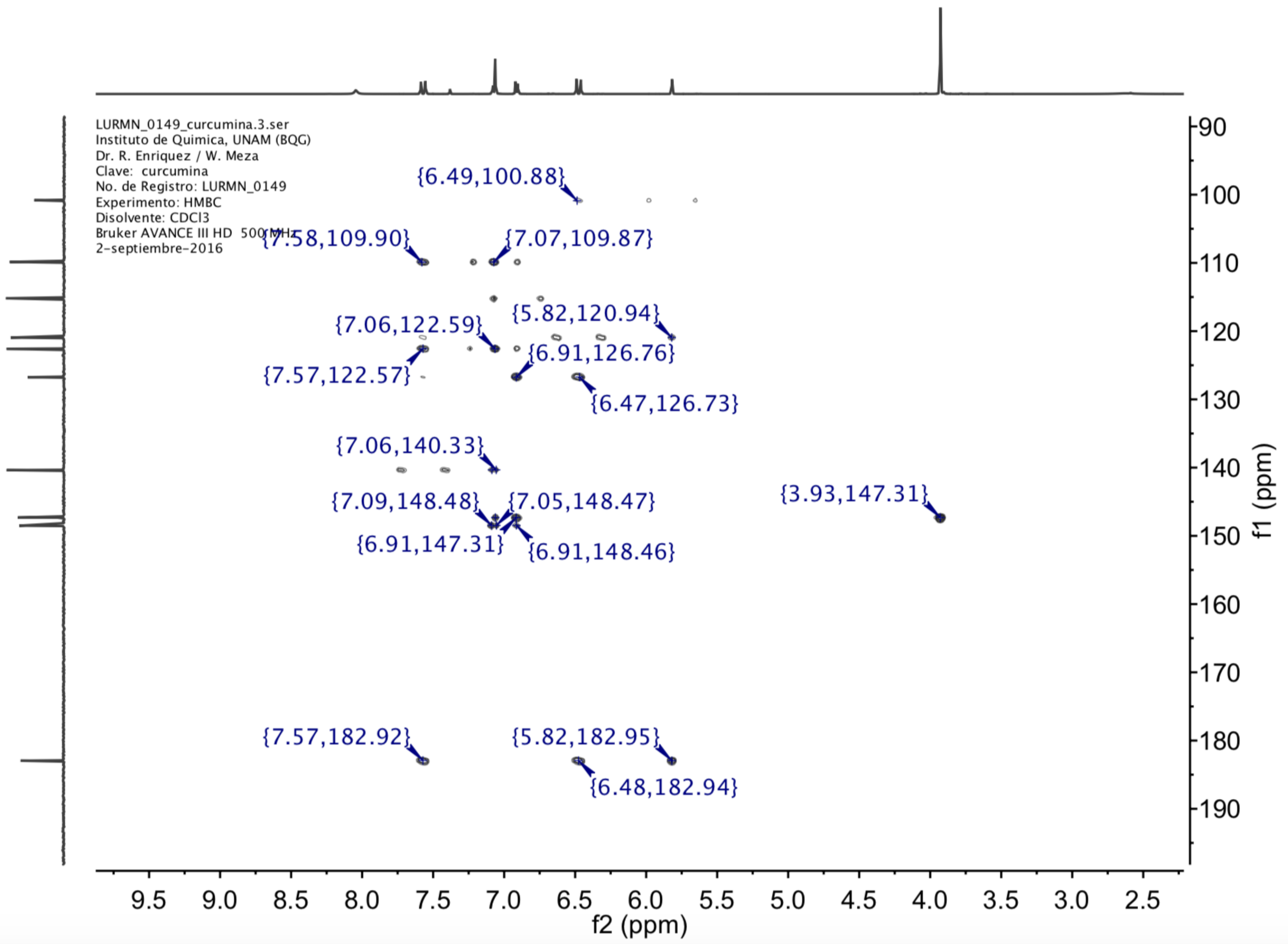

Figure S6: HMBC of curcumin (**1**).

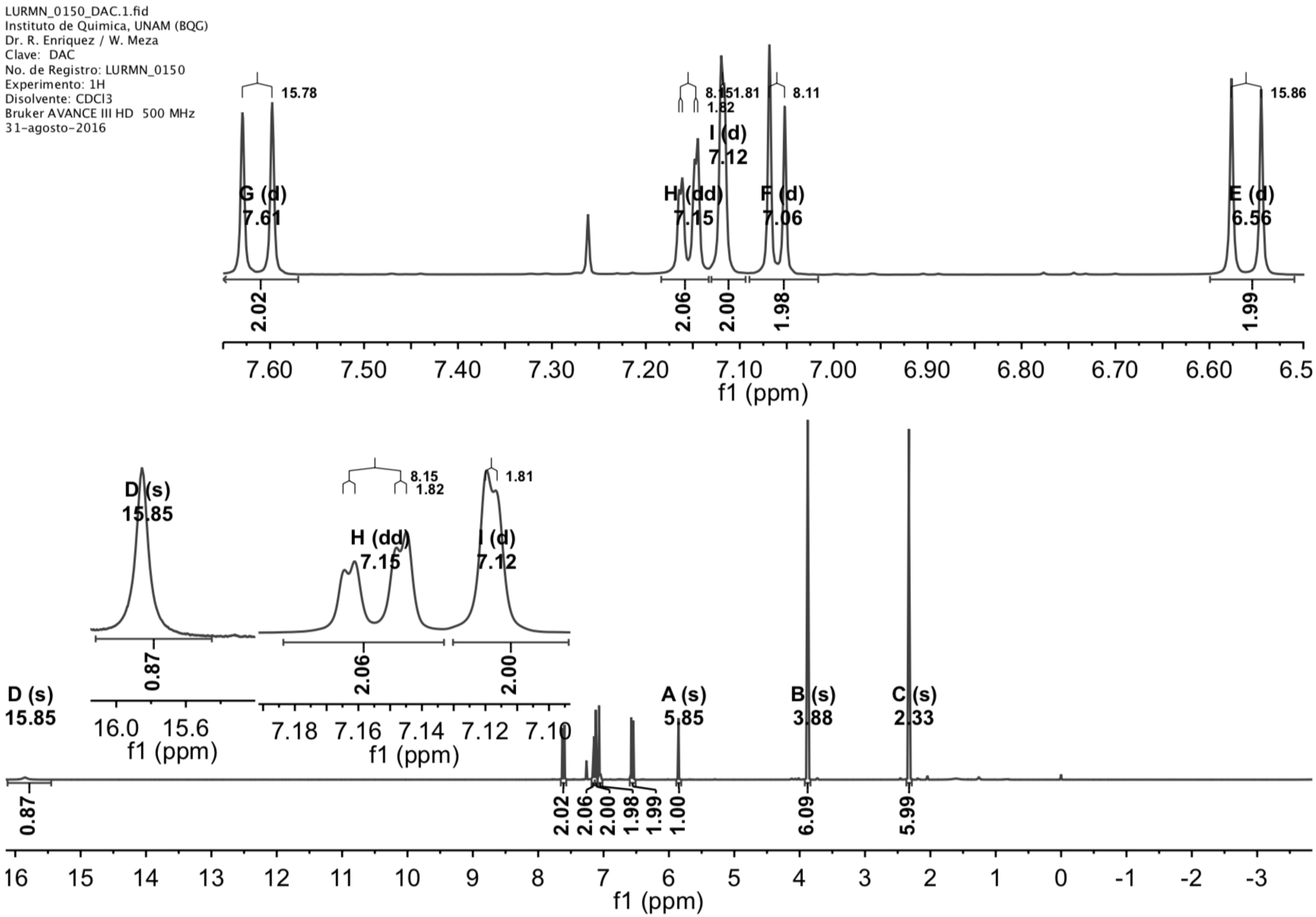

Figure S7: ^{1}H-NMR of diacetylcurcumin (**2**).

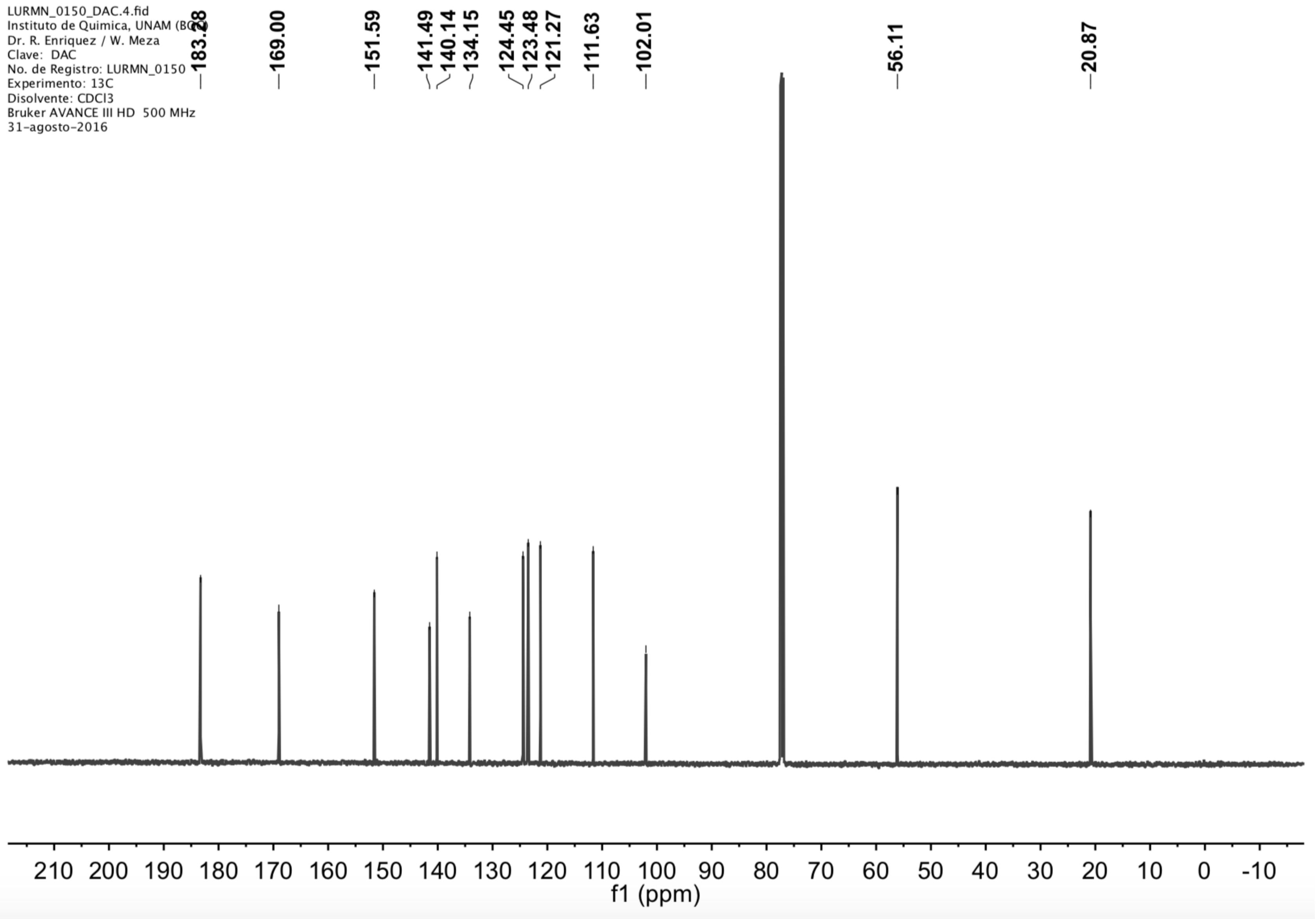

Figure S8: ^{13}C-NMR of diacetylcurcumin (**2**).

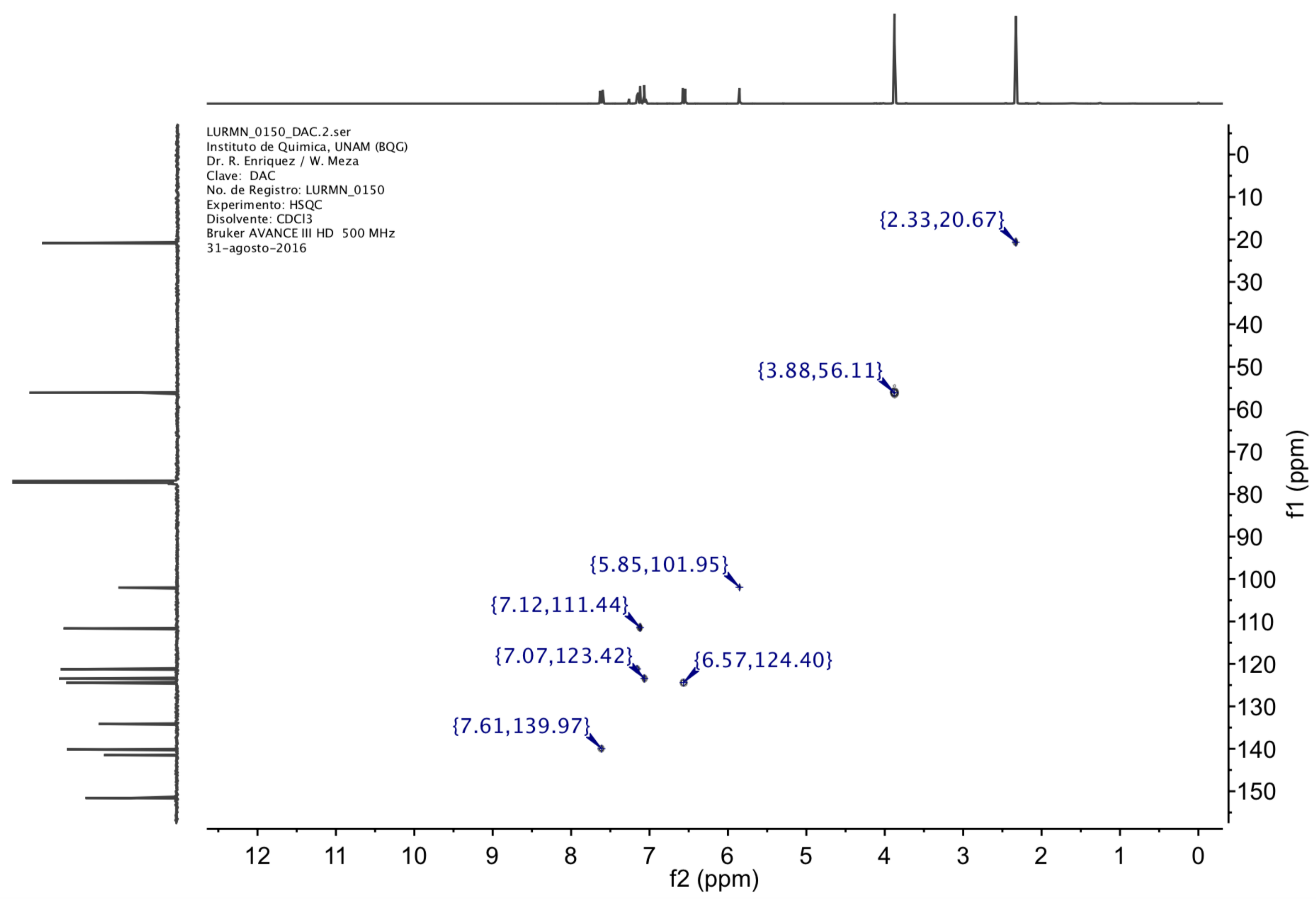

Figure S9: HSQC of diacetylcurcumin (**2**).

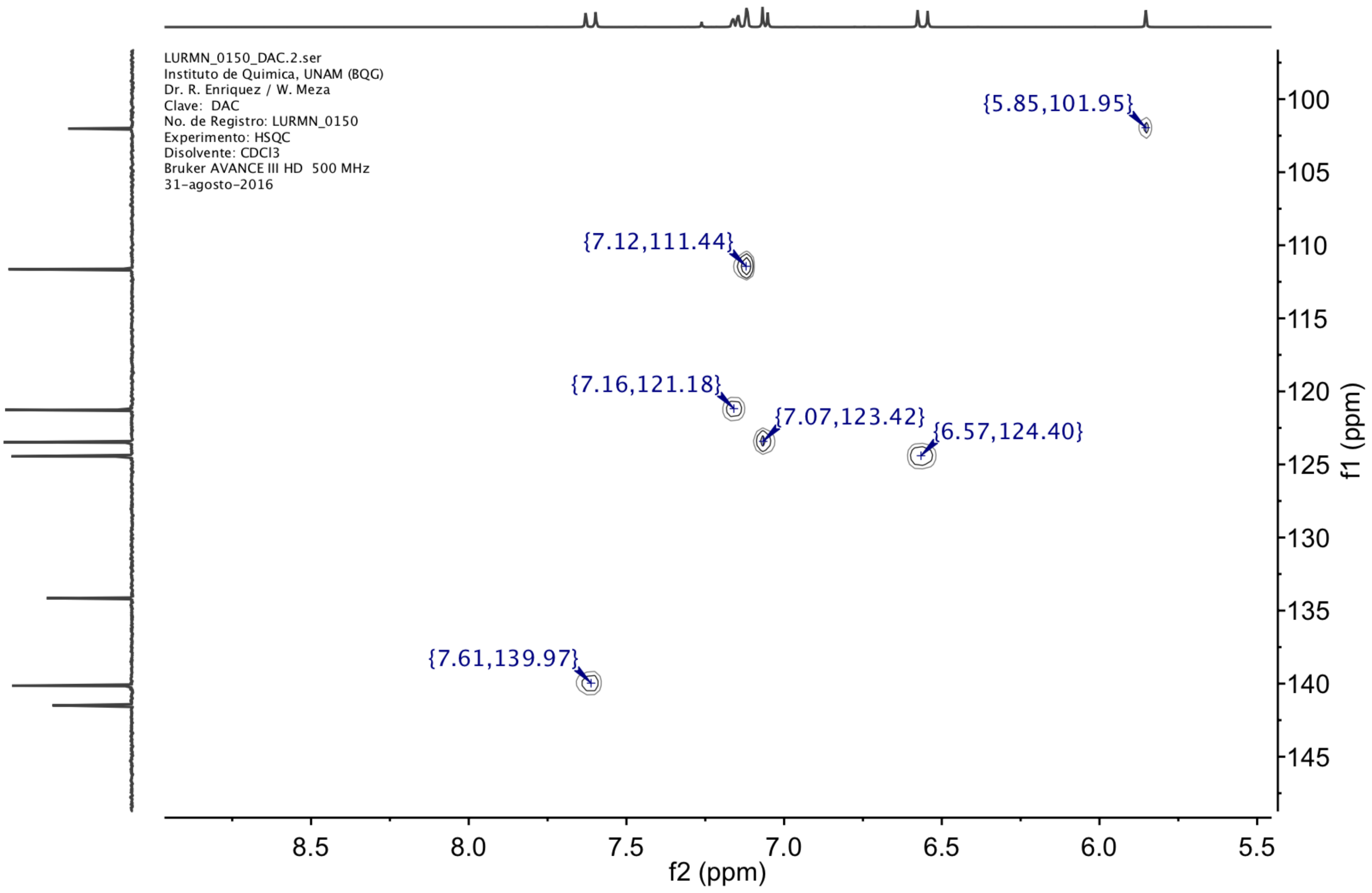

Figure S10: HSQC of diacetylcurcumin (**2**).

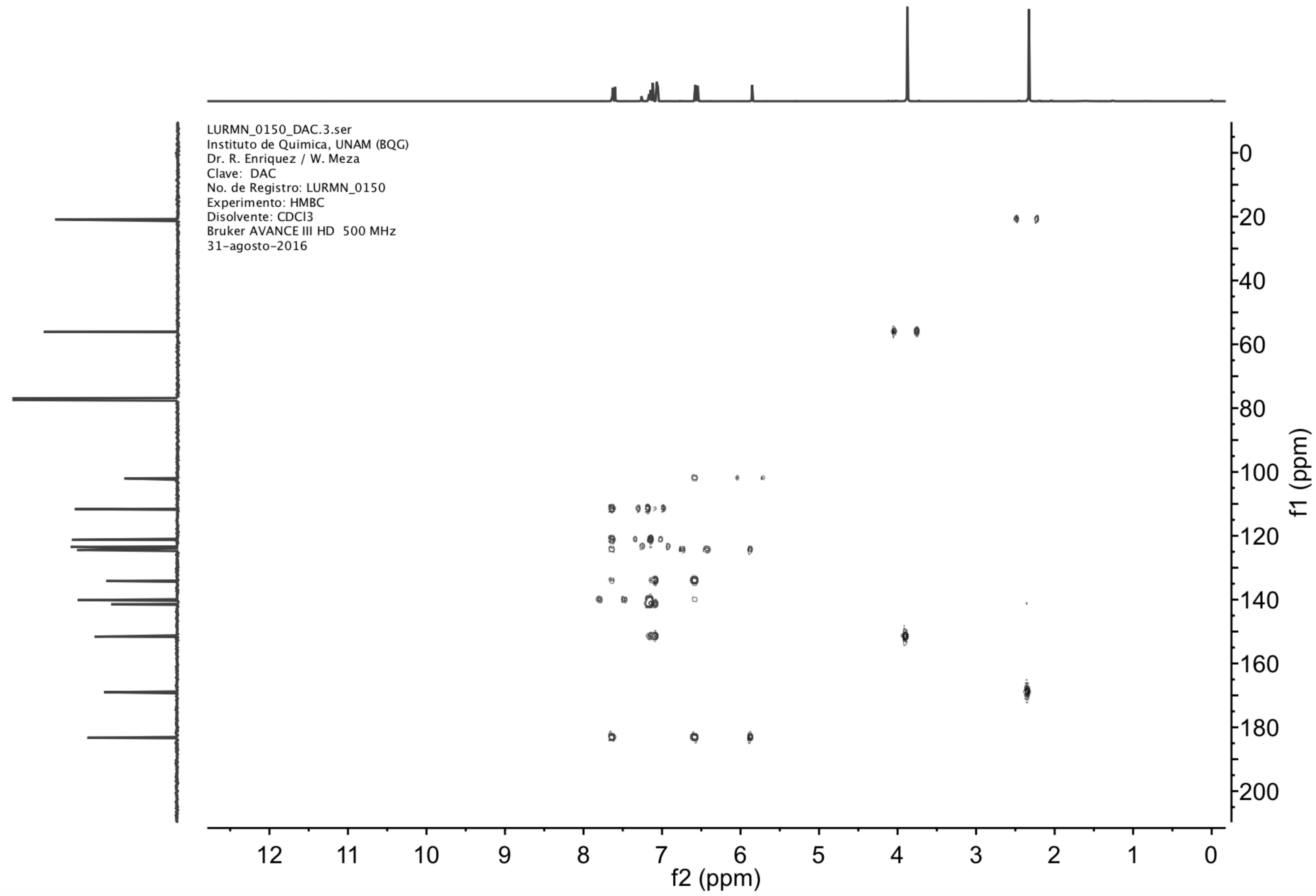

Figure S11: HMBC of diacetylcurcumin (**2**).

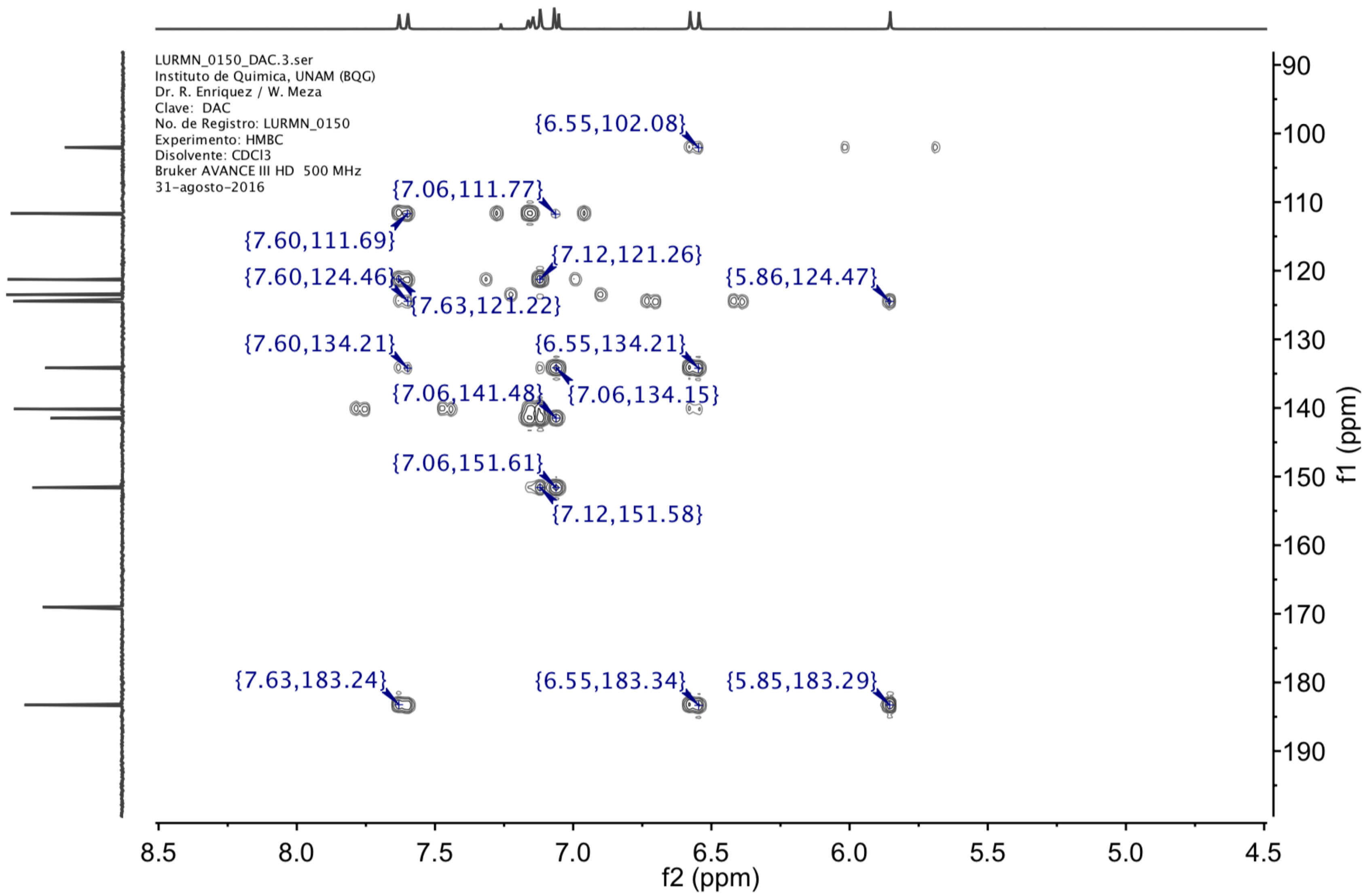

Figure S12: HMBC of diacetylcurcumin (**2**).
